# New advances in *DPYD* genotype and risk of severe toxicity under capecitabine

**DOI:** 10.1371/journal.pone.0175998

**Published:** 2017-05-08

**Authors:** Marie-Christine Etienne-Grimaldi, Jean-Christophe Boyer, Christophe Beroud, Litaty Mbatchi, André van Kuilenburg, Christine Bobin-Dubigeon, Fabienne Thomas, Etienne Chatelut, Jean-Louis Merlin, Frédéric Pinguet, Christophe Ferrand, Judith Meijer, Alexandre Evrard, Laurence Llorca, Gilles Romieu, Philippe Follana, Thomas Bachelot, Loic Chaigneau, Xavier Pivot, Véronique Dieras, Rémy Largillier, Mireille Mousseau, Anthony Goncalves, Henri Roché, Jacques Bonneterre, Véronique Servent, Nadine Dohollou, Yann Château, Emmanuel Chamorey, Jean-Pierre Desvignes, David Salgado, Jean-Marc Ferrero, Gérard Milano

**Affiliations:** 1 Centre Antoine Lacassagne, Nice, France; 2 CHU de Nîmes, Nîmes, France; 3 Aix-Marseille University, INSERM UMR S910, GMGF, Marseille, France; 4 APHM Hôpital Timone, Laboratoire de Génétique Moléculaire, Marseille, France; 5 Faculté de Pharmacie de Montpellier, Montpellier, France; 6 Laboratory Genetic Metabolic Diseases, Academic Medical Center, Amsterdam,The Netherlands; 7 Institut de Cancérologie de l’Ouest, MMS EA 2160, Université de Nantes, Nantes, France; 8 Institut Claudius-Regaud, CRCT, Université de Toulouse, Inserm, UPS, Toulouse, France; 9 Institut de Cancérologie de Lorraine, UMR CNRS 7039 CRAN, Université de Lorraine, Nancy, France; 10 Centre Paul Lamarque, Montpellier, France; 11 CHU de Besançon, Besançon, France; 12 Centre Léon Bérard, Lyon, France; 13 Institut Curie, Paris, France; 14 Centre Azuréen de Cancérologie, Mougins, France; 15 CHU de Grenoble, Grenoble, France; 16 Institut Paoli Calmettes, Marseille, France; 17 Centre Oscar Lambret, Lille, France; 18 Polyclinique Nord Aquitaine, Bordeaux, France; CNR, ITALY

## Abstract

**Background:**

Deficiency in dihydropyrimidine dehydrogenase (DPD) enzyme is the main cause of severe and lethal fluoropyrimidine-related toxicity. Various approaches have been developed for DPD-deficiency screening, including *DPYD* genotyping and phenotyping. The goal of this prospective observational study was to perform exhaustive exome *DPYD* sequencing and to examine relationships between *DPYD* variants and toxicity in advanced breast cancer patients receiving capecitabine.

**Methods:**

Two-hundred forty-three patients were analysed (88.5% capecitabine monotherapy). Grade 3 and grade 4 capecitabine-related digestive and/or neurologic and/or hemato-toxicities were observed in 10.3% and 2.1% of patients, respectively. *DPYD* exome, along with flanking intronic regions 3’UTR and 5’UTR, were sequenced on MiSeq Illumina. DPD phenotype was assessed by pre-treatment plasma uracil (U) and dihydrouracil (UH2) measurement.

**Results:**

Among the 48 SNPs identified, 19 were located in coding regions, including 3 novel variations, each observed in a single patient (among which, F100L and A26T, both pathogenic *in silico*). Combined analysis of deleterious variants *2A, I560S (*13) and D949V showed significant association with grade 3–4 toxicity (sensitivity 16.7%, positive predictive value (PPV) 71.4%, relative risk (RR) 6.7, p<0.001) but not with grade 4 toxicity. Considering additional deleterious coding variants D342G, S492L, R592W and F100L increased the sensitivity to 26.7% for grade 3–4 toxicity (PPV 72.7%, RR 7.6, p<0.001), and was significantly associated with grade 4 toxicity (sensitivity 60%, PPV 27.3%, RR 31.4, p = 0.001), suggesting the clinical relevance of extended targeted *DPYD* genotyping. As compared to extended genotype, combining genotyping (7 variants) and phenotyping (U>16 ng/ml) did not substantially increase the sensitivity, while impairing PPV and RR.

**Conclusions:**

Exploring an extended set of deleterious *DPYD* variants improves the performance of *DPYD* genotyping for predicting both grade 3–4 and grade 4 toxicities (digestive and/or neurologic and/or hematotoxicities) related to capecitabine, as compared to conventional genotyping restricted to consensual variants *2A, *13 and D949V.

## Introduction

Since its launch in 1998, the 5FU oral prodrug capecitabine has gradually become a major drug and is currently considered as a standard of care for advanced breast cancer. Capecitabine is ultimately metabolized by thymidine phosphorylase which produces 5FU at target cell level. Next, intracellularly-produced 5FU enters either the anabolic or the catabolic route. Most 5FU is deactivated into fluorodihydrouracil by ubiquitous dihydropyrimidine dehydrogenase (DPD), the rate-limiting enzyme of 5FU catabolism, expressed in various human tissues as well as in human cancer cells [1,2]. Consequently, any DPD activity variation within tumor or normal cells may have a major repercussion on availability of 5FU for anabolism, and thus may significantly impact capecitabine pharmacodynamics. DPD deficiency may be considered as the major cause of capecitabine toxicity, and more generally fluoropyrimidine-related toxicity risk [3–6]. Accordingly, the wide inter-patient variability of DPD enzyme activity measured in peripheral blood mononuclear cells (PBMC) is significantly correlated to systemic 5FU clearance in patients receiving i.v. 5FU [3,7]. Of note, breast cancer treatment with capecitabine is particularly concerned since PBMC-DPD activity has been shown to be lower in women as compared to men [7], in line with the observation that women are particularly prone to suffer from fluoropyrimidine toxicity [8]. Importantly, cases of lethal toxicity have been reported in patients with marked DPD deficiency after standard 5FU [9] or capecitabine administration [10–12].

DPD is encoded by *DPYD*, a large gene spanning 950 kb on chromosome 1p22 (23 exons comprising 4399 nucleotides) [13]. DPD activity is controlled at both transcriptional and post-transcriptional levels. Post-transcriptional regulation of DPD involved microRNAs miR-27a and miR-27b [14]. At transcriptional level, more than 200 polymorphisms have been identified in *DPYD* coding regions. *In vitro* studies have demonstrated that only a few *DPYD* polymorphisms have a significant deleterious impact on enzymatic activity, while even fewer are associated with proficient (i.e. elevated) enzyme activity [15,16]. It has been clearly demonstrated that *DPYD* deleterious variants *2A (c.1905+1G>A), D949V (c.2846A>T), and *13 (I560S, c.1679T>G) are relevant predictors of fluoropyrimidine-related toxicities [6,17–19]. From a prospective trial conducted on 2594 colon cancer patients receiving adjuvant 5FU-based chemotherapy [20], the combined sensitivity of the variants *2A, *13 and D949V to predict grade 3–4 5FU-related toxicity is only 5.3% (33% toxicity, 2.2% of patients carrying a variant). The scarcity of these three *DPYD* variations explains this very low sensitivity: considering at best 4% of patients carrying one of these three pathological variants and a prevalence of severe toxicity at 10%, a genetic test based on these 3 variants cannot have a sensitivity greater than 40%. One could thus expect to improve sensitivity by identifying additional relevant variants, by combining genotyping and phenotyping approaches, or by focusing on the less frequent, most relevant grade 4 toxicities.

Despite decades of literature data on *DPYD* pharmacogenetics, very few clinical prospective studies have reported full *DPYD* exome sequencing in patients treated by fluoropyrimidines [21–24], and most of these studies were based on small population subsets. The French GPCO-Unicancer group recently conducted a prospective observational study on 303 advanced breast cancer patients receiving capecitabine to assess the impact of pre-treatment DPD and CDA phenotype along with a limited number of targeted variants in DPYD (*2A, *13, *D949V)*, *TYMS* and *MTHFR* genes, on capecitabine toxicity and efficacy [25]. DPD phenotyping was based on pretreatment measurement of plasma uracil (U) and dihydrouracil (UH2). We presently report a genomic-based complementary study conducted in 243 patients for whom full sequencing of *DPYD* exome, along with flanking intronic regions, was performed in order to examine relationships between *DPYD* variants and both DPD phenotype and severe capecitabine-related toxicity.

## Patients and methods

### Patients

**Fig 1:**
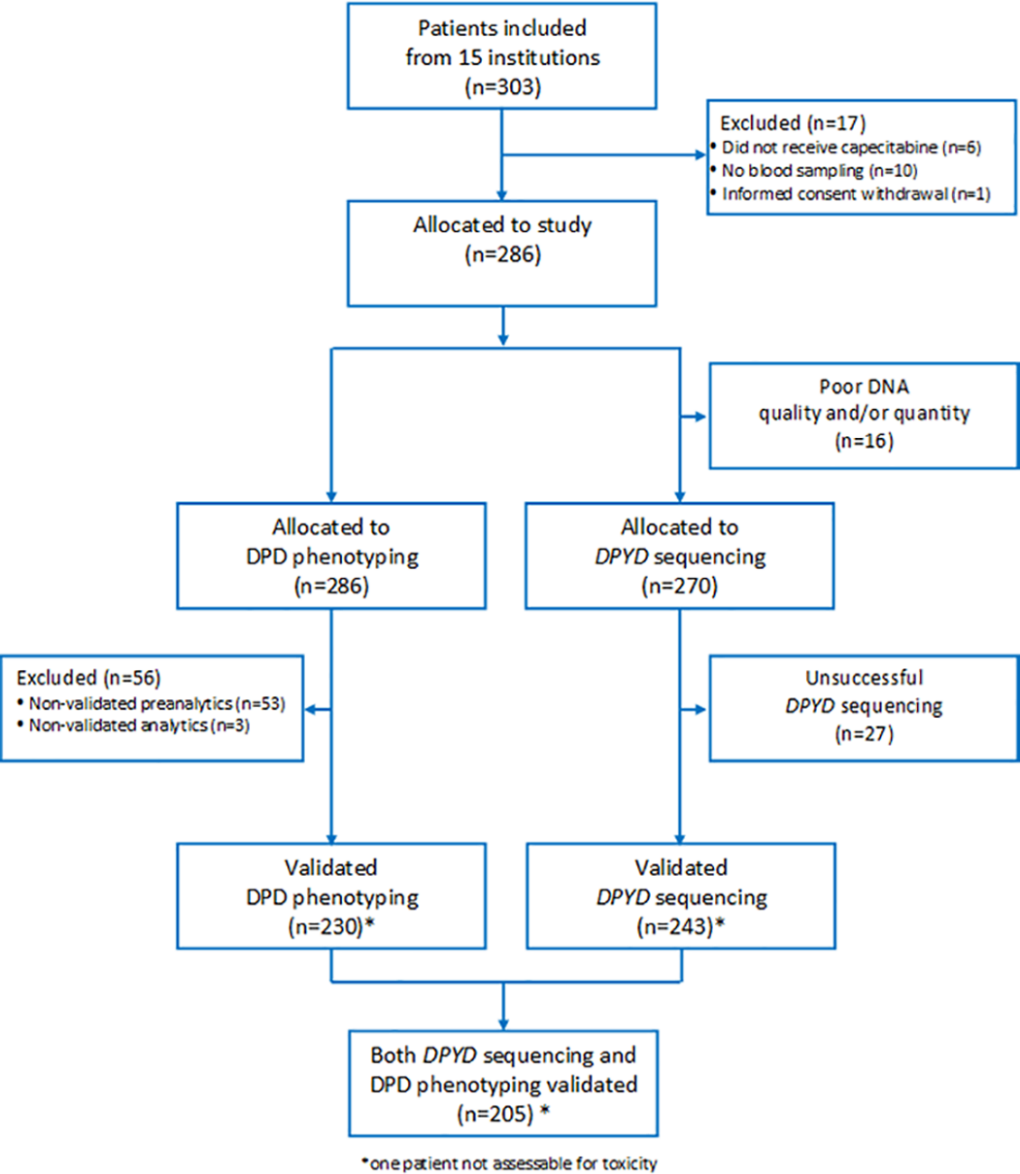
CONSORT diagram.

This prospective observational study (Eudract 2008-004136-20) was conducted on 303 advanced breast cancer patients included between February 2009 and February 2011 in 15 French institutions. This study was approved by the "Comité de Protection des Personnes—Méditerranée Sud V" (approval number CPP 08.067). Written informed consent was obtained for each patient. Among the 303 initially-included patients, 17 were not allocated to the study; DNA was not available for 16 patients and *DPYD* sequencing was unsuccessful (poor quality score) for 27 patients (**Fig 1**, CONSORT Diagram). Thus, a total of 243 patients were included in the present analysis (12 recruiting centers). Inclusion criteria were women above 18-years-old with histologically-proven advanced breast cancer starting capecitabine treatment alone or in combination with anti-angiogenic therapy, whatever previous metastatic treatment lines were delivered, provided they did not include a fluoropyrimidine. Previous adjuvant treatment with fluoropyrimidine was allowed. Exclusion criteria included concomitant chemotherapy or lapatinib therapy, uncontrolled brain metastasis, uncontrolled chronic illness or infection, life expectancy lower than 3 months, cardiac failure or hypoxic respiratory failure. Capecitabine treatment was administered orally in two daily doses for 14 days, followed by 7 days off (day 1 = day 21). The capecitabine dose was left to the discretion of the physician. One to 15 days before starting treatment, 15 ml of blood were taken in the morning (8 am—11 am) for U and UH2 plasma analysis and *DPYD* genotyping. Toxicity (CTCAE v3 criteria) was assessed over cycles 1–2 (up to 21 days after the end of the 2^nd^ cycle) in 242 patients.

### Biological analyses

#### *DPYD* sequencing

DNA from total blood (10 ml) was extracted in one out of the 7 participating laboratories according to local routine procedures. DNA (1 μg required for *DPYD* sequencing) was sent to Integragen (Evry, France) where sequencing was performed. The 23 exons of *DPYD* gene, along with flanking intronic regions (20 bp), 3’UTR and part of 5’UTR (580 bp upstream transcription initiation) were sequenced on MiSeq Illumina (PCR multiplex, 2x150 nt paired-end sequencing). Amplicons were sequenced in both directions. Obtained sequences were mapped to the human reference assembly genome HG19 and variant calling was performed on the NM_000110 reference transcript. SNPs alignment and variant calling were performed with the Illumina pipeline CASAVA1.8 using ELANDv2 algorithm. On average, 97% of target sequences were successfully covered with a mean depth at 1200X. Validated DNA samples passed quality scores (Q30 quality filter >90%, depth >15X). Indels were identified using the SNAP/GATK pipeline [26]. Local realignment, base recalibration and haplotype caller were performed using the GATK tool. INDELs with a quality score >50 and a depth >10X were selected. Pairwise linkage disequilibria (LD) between bi-allelic *DPYD* variants were measured by D’ and D’/LOD calculated on Haploview software v4.2.

#### *In silico* functional prediction

*In silico* pathogenicity prediction of coding variants was performed with the UMD-Predictor system [27]. Impact of exonic and intronic variations on splicing signal types was predicted with the Human Splicing Finder system [28].

### DPD phenotyping

Blood (5 ml) was immediately placed in an ice-bath, centrifuged at +4°C for 15 min and frozen plasma was stored at -80°C. In order to mimic routine DPD-deficiency screening and ensure robustness, frozen plasmas were sent to one of 3 measuring laboratories (according to geographic location). Depending on the laboratory, solid-phase or liquid-liquid extraction followed by HPLC analysis (UV detection) of uracil (U) and dihydrouracil (UH2) was performed [29–31]. Limit of quantification was 7 to 25 ng/ml for UH2 and 3 to 6 ng/ml for U, depending on the laboratory. A common external quality control (N = 52 aliquots) was shared across the 3 laboratories. For UH2, 100% of control values were within +/- 15% of the mean value (92.2 ng/ml) and for U 98% of control values were within +/- 15% of the mean value (7.9 ng/ml) with one control value at +22%. Regarding the UH2/U ratio, 94% of control values were within +/- 15% of the mean value (11.7 ng/ml), with 3 values at -25%, -19% and +19%, respectively. Possible influence of measuring laboratories and recruiting centers on U and UH2/U concentrations was checked. Plasma U and UH2/U from patients were not significantly different between measuring laboratories. In contrast, comparison between recruiting centers showed that, in one institution U and UH2/U from patients differed significantly from other institutions (ANOVA tests: p<0.001 for both U and UH2/U), suggesting a pre-analytical deviation. In order to accurately examine the impact of *DPYD* variants on U and UH2/U plasma concentrations, we excluded phenotype data from this recruiting center. In total, two hundred and five patients with validated *DPYD* sequencing were thus considered for phenotype analysis.

### Statistics

The initial required number of 300 patients was based on the hypothesis that 35% of “at-risk patients” will develop grade 3–4 capecitabine-related toxicity *versus* 12% in the group of patients without risk (unilateral test, alpha = 0.05, beta = 0.10, relative risk = 2.9), with “at-risk patients” defined as those exhibiting a DPD-deficient phenotype. Hypothesis for DPD-deficient phenotype was UH2/U below 15^th^ percentile or U above the 85^th^ percentile. Considering patients with validated phenotyping data, actual study power was able to detect a relative risk (RR) of 3.50. The influence of each individual variant on DPD phenotype and capecitabine-related toxicity was assessed for *DPYD* variants present in at least 3 patients, using the non-parametric Mann-Whitney test and the Fisher Exact test, respectively. *DPYD* genotypes were considered as binary variables (wt/wt *vs* wt/var+var/var). Capecitabine-related toxicity included hematotoxicity, digestive toxicity and neurotoxicity. Sensitivity, specificity, positive predictive value (PPV), negative predictive value (NPV) and relative risk (RR) associated with DPD-deficiency screening approaches were computed. Sensitivity is defined as the proportion of patients found to be positive for DPD-deficiency among those experiencing toxicity. Specificity is defined as the proportion of patients without DPD-deficiency among those without toxicity. PPV is defined as the proportion of patients experiencing toxicity among those positive for the test. NPV is defined as the proportion of patients without toxicity among those negative for the test. RR is defined as the ratio of the toxicity risk in patients positive for the test to that in patients negative for the test. For the approach combining genotype and phenotype, we considered positivity of either one of the two approaches. All tests were two-sided and were not corrected for multiple testing. All p values ≤ 0.05 were reported. Statistics were performed on SPSS software (v15).

## Results

### Patient characteristics and toxicity

**Table 1**describes patients’ characteristics. Mean age was 61.2 years (range 30–88). 88.5% of patients received capecitabine as monotherapy. Mean capecitabine dose at cycle 1 was 1942 mg/m^2^/day (median 1964, range 65–2590). Capecitabine-related digestive toxicity, hematotoxicity and neurotoxicity, grade (G) 3–4 was observed in 12.4% of patients (30 patients), and G4 was observed in 5 patients (2.1%), including one toxic death (**S1 Table**). Hand-foot syndrome G3 was observed in 9.5% of patients (no G4). Toxicity was not related to PS status, patient age, renal function (creatinine clearance), capecitabine treatment line, previous adjuvant fluoropyrimidine treatment, or capecitabine dose at first cycle. **Table 2**details the profile of patients presenting G4-5 toxicity. A patient with toxic death (80-year-old) presented presented G4 thrombopenia, G4 diarrhea, G4 renal failure, G5 dyspnea and hypovolemic shock, 20 days after starting capecitabine monotherapy. This patient had lung and cutaneous metastases (PS 0, history of arterial hypertension), exhibited uracilemia at 16.7 ng/ml, UH2/U ratio at 6.5, was heterozygous for D949V polymorphism and carried 2 other variations in *DPYD* 3’UTR.

**Table 1:** Patient and treatment characteristics (N = 243).

	N	%
**Performance status** 0 1 2 3 Unknown	78 65 21 4 75	32.1 26.7 8.6 1.6 30.9
**Previous adjuvant fluoropyrimidine** No Yes (5FU/capecitabine)	153 90 (89/1)	63% 37%
Metastasis site* Bone Liver Lung Lymph node Cutaneous Brain Others	161 127 95 62 32 9 12	66.3 52.3 39.1 25.5 13.2 3.7 4.9
**Capecitabine treatment** Monotherapy Concurrent bevacizumab Concurrent trastuzumab Concurrent lapatinib**	215 18 7 3	88.5 7.4 2.9 1.2
**Capecitabine line** 1^st^ line 2^nd^ line 3^rd^ line ≥ 4^th^ line	70 87 61 25	28.8 35.8 25.1 10.3
**Number of Capecitabine cycles** ≥ 1 cycle ≥ 2 cycles ≥ 3 cycles	243 224 208	100 92.2 85.6

* sum greater than 243 patients due to multiple metastases sites.

** these 3 patients (protocol violation) were kept in final analysis (none developed hematotoxicity, one developed digestive toxicity (grade 2) and 2 developed cutaneous toxicity (grade 3)).

**Table 2:** Profile of patients with grade 4–5 toxicity.

	Patient #1	Patient #2	Patient #3	Patient #4	Patient #5
Toxicity*	Toxic death** (cycle 1)	G4 anemia G4 thrombopenia G3 neutropenia (cycle 1)	G4 thrombopenia G3 asthenia (cycle 1)	G3 neurotoxicity (cycle 1) G4 thrombopenia G4 neutropenia G4 leucopenia (cycle 2)	G4 diarrhea (cycle 2)
**Mean capecitabine dose intensity at cycle 1** (mg/m^2^/day)	1530	2030	1790	2490	2170
**PS at inclusion**	0	1	na	2	0
**Pre-treatment UH2/U** **Pre-treatment U** (ng/ml)	6.5 16.7	14.0 12.9	13.5 22.0	na na	na na
***DPYD* variants**					
c.-477T>G	wt/wt	wt/wt	VAR/wt	wt/wt	wt/wt
C29R	wt/wt	wt/wt	VAR/wt	wt/wt	wt/wt
F100L	wt/wt	wt/wt	wt/wt	VAR/wt	wt/wt
c.483+837A>G ^**§**^	wt/wt	wt/wt	VAR/wt	wt/wt	wt/wt
c.483+1342T>A ^**§**^	wt/wt	wt/wt	VAR/wt	wt/wt	wt/wt
c.483+1344T>A ^**§**^	wt/wt	wt/wt	VAR/wt	wt/wt	wt/wt
M166V ^**§**^	wt/wt	wt/wt	VAR/wt	wt/wt	wt/wt
c.1129-15T>C ^**§**^	wt/wt	wt/wt	VAR/wt	wt/wt	wt/wt
S492L	wt/wt	wt/wt	wt/wt	wt/wt	VAR/wt
D949V	VAR/wt	wt/wt	wt/wt	wt/wt	wt/wt
c.*274T>C ^**§§**^	wt/wt	VAR/VAR	wt/wt	wt/wt	wt/wt
c.*432T>A	wt/wt	wt/wt	VAR/wt	wt/wt	wt/wt
c.*768G>A ^**§§**^	VAR/VAR	wt/wt	VAR/VAR	VAR/VAR	wt/wt
c.*780C>T ^**§§**^	VAR/wt	wt/wt	VAR/VAR	VAR/wt	wt/wt

* All grade 3–4 toxicities.

** See Results section for details.

^**§**^ these 5 variants were in linkage disequilibria (see S2 Fig).

^**§§**^ these 3 variants were in linkage disequilibria (see S2 Fig).

na means not available.

These 5 patients all received capecitabine as monotherapy.

### Description of *DPYD* variants (Table 3)

**Table 3:** Description of *DPYD* variations along with *in silico* / *in vitro* functionality.

SNP or INDEL position (or rs if any)	Nucleotide change and nomenclature alias (if any)	Location	AA change	*In silico* pathogenicity prediction (impact on splicing signal)	*In vitro* functionality [15] / [16]	wt/wt	Case number var/wt	var/var	MAF (%)	Significant association with deficient phenotype*	Significant association with increased toxicity**
**rs145438244**	c.-672T>C	5-UTR				241	1	0	0.2	nt	nt
**rs61787828**	c.-477T>G	5-UTR				214	27	1	6.0	NS	NS
98386496	c.-18G>A	5-UTR				241	1	0	0.2	na, nt	nt
98348989	c.40-69_40-59del	Intron 1		(No impact on splicing)		242	1	0	0.2	nt	nt
98348894	c.76G>A	Exon 2	A26T	Pathogenic (ESE site broken)	- / -	241	1	0	0.2	nt	nt
**rs1801265**	c.85T>C (*9A)	Exon 2	C29R	Benign (ESE site broken, new ESS)	- / Slightly deficient	161	74	7	18.2	NS	No***
**rs371587702**	c.194C>T	Exon 3	T65M	Pathogenic (ESE site broken)	Benign / -	241	1	0	0.2	nt	nt
98205969	c.300C>A	Exon 4	F100L	Pathogenic (ESE site broken)	F100[FS] very deficient / -	241	1	0	0.2	na, nt	nt
**rs56276561**	c.483+18G>A ^#^	Intron 5		(No impact on splicing)		238	4	0	0.8	NS	NS
98186503	c.483+563T>C	Intron 5		(No impact on splicing)		241	1	0	0.2	nt	nt
98186337	c.483+729G>A	Intron 5		(No impact on splicing)		241	1	0	0.2	nt	nt
**rs56066952**	c.483+834A>G	Intron 5		(No impact on splicing)		240	2	0	0.4	nt	nt
**rs55684412**	c.483+837A>G	Intron 5		(No impact on splicing)		195	44	3	10.3	NS	NS
98185786	c.483+1280A>G	Intron 5		(No impact on splicing)		242	1	0	0.2	nt	nt
**rs61786599**	c.483+1342T>A	Intron 5		(No impact on splicing)		202	40	1	8.6	NS	NS
**rs61786598**	c.483+1344T>A	Intron 5		(No impact on splicing)		192	48	3	11.1	NS	NS
98185721	c.483+1345_483+1354del	Intron 5		(No impact on splicing)		242	1	0	0.2	nt	nt
**rs199919864**	c.483+1346A>T	Intron 5		(No impact on splicing)		242	1	0	0.2	na, nt	nt
98185720	c.483+1345_483+1346dup	Intron 5		(No impact on splicing)		241	2	0	0.4	nt	nt
98185711	c.483+1354_483+1355insAA	Intron 5		(No impact on splicing)		242	1	0	0.2	nt	nt
98185705	c.483+1360_483+1361dup	Intron 5		(No impact on splicing)		233	10	0	2.1	NS	NS
**rs75848562**	c.483+1366A>G	Intron 5		(No impact on splicing)		232	11	0	2.3	NS	NS
**rs142148197**	c.483+1689G>A	Intron 5		(No impact on splicing)		242	1	0	0.2	nt	nt
**rs2297595**	c.496A>G	Exon 6	M166V	Benign (New ESS site and cryptic acceptor splice site)	Proficient / Slightly deficient	198	42	3	9.9	NS	NS
98060744	c.851-22T>C	Intron 8		(No impact on splicing)		241	1	0	0.2	nt	nt
**rs183385770**	c.1025A>G	Exon 10	D342G	Pathogenic (ESE site broken, new ESS site)	D342N very deficient / -	241	1	0	0.2	nt	nt
98058849	c.1053T>C	Exon 10	A351A	Benign (No impact on splicing)		241	1	0	0.2	nt	nt
98058804	c.1098C>T	Exon 10	G366G	Probably pathogenic (New ESS site)		241	1	0	0.2	na, nt	nt
**rs56293913**	c.1129-15T>C	Intron 10		(No impact on splicing)		191	46	5	11.6	NS	NS
**rs61622928**	c.1218G>A	Exon 11	M406I	Benign (No impact on splicing)	Benign / Benign	242	1	0	0.2	nt	nt
**rs56038477**	c.1236G>A ^#^	Exon 11	E412E	Benign (No impact on splicing)		239	4	0	0.8	NS	NS
**rs72549304**	c.1475C>T	Exon 12	S492L	Pathogenic (New ESS site)	Very deficient / -	241	1	0	0.2	na, nt	nt
**rs199469537**	c.1524 +16C>A	Intron 12		(No impact on splicing)	-	241	1	0	0.2	na, nt	nt
**rs1801158**	c.1601G>A (*4)	Exon 13	S534N	Probably benign (New ESS site)	- / Slightly deficient	234	8	0	1.7	NS	NS
**rs1801159**	c.1627A>G	Exon 13	I543V	Benign (No impact on splicing)	- / Benign	161	73	8	18.4	NS	NS
**rs55886062**	c.1679T>G (*13)	**Exon 13**	**I560S**	Pathogenic (No impact on splicing)	Very deficient / -	242	1	0	0.2	na, nt	nt
97981200	c.1740+82del	Intron 13		(No impact on splicing)		242	1	0	0.2	nt	nt
**rs59086055**	c.1774C>T	Exon 14	R592W	Pathogenic (New ESS site)	Very deficient / -	241	1	0	0.2	nt	nt
**rs17376848**	c.1896T>C	Exon 14	F632F	Benign (New ESS site)		227	15	1	3.5	NS	NS
**rs3918290**	c.1905+1G>A (*2A)	**Splice intron 14**		Pathogenic (Alteration of the donor site)	Very deficient /	240	3	0	0.6	**Yes**	**Yes**
rs369990607	c.1905+17A>G	Intron 14		(No impact on splicing)		242	1	0	0.2	nt	nt
**rs12078940**	c.1906-24G>A	Intron 14		(No impact on splicing)		241	1	0	0.2	nt	nt
97771825	c.2087G>A	Exon 17	R696H	Probably pathogenic (No impact on splicing)	- / -	241	1	0	0.2	nt	nt
**rs55846082**	c.2179+28C>T	Intron 17		(No impact on splicing)		241	1	0	0.2	nt	nt
**rs138637410**	c.2179+29G>A	Intron17		(No impact on splicing)		241	1	0	0.2	na, nt	nt
**rs1801160**	c.2197G>A (*6)	Exon 18	V732I	Benign (No impact on splicing)	V732G benign / V732I Slightly deficient	226	16	0	3.3	NS	NS
**rs67376798**	**c.2846A>T**	**Exon 22**	**D949V**	Probably pathogenic (ESE site broken, new ESS site)	Moderately deficient / Moderately deficient	240	3	0	0.6	**Yes**	**Yes**
**rs56160474**	c.*274T>C	3-UTR				158	75	9	19.2	NS	NS
**rs188501488**	c.*432T>A	3-UTR				240	2	0	0.4	nt	nt
**rs291592**	c.*768G>A	3-UTR				85	122	35	39.7	NS	NS
**rs291593**	c.*780C>T	3-UTR				151	78	13	21.5	NS	NS
**rs17470762**	c.*900T>C	3-UTR				222	19	1	4.3	NS	NS
**rs41285690**	c.*1062A>G	3-UTR				238	4	0	0.8	NS	NS
97543343	c.*1189G>A	3-UTR				241	1	0	0.2	nt	nt

SNP and INDEL positions are given relative to genome build 37 HG19 (reference = nucleotide A of the translation initiation codon ATG).

*In silico* pathogenicity of coding variants was predicted using UMD-Predictor system [27]. The potential impact of exonic and intronic variations on splicing signal types was predicted using Human Splicing Finder system [28].

*In vitro* functionality derived from two published *in vitro* functional studies [15,16] reporting DPD enzyme activity of missense *DPYD* variants transgenically expressed in mammalian cells. In both studies, deficiency and proficiency were based on statistical comparison relative to wild-type DPD activity (100% activity). p value considered statistically significant was 0.05 in the study by Offer [15] and 0.001 in the study by van Kuilenburg [16]. Statistically significant DPD deficiency was classified as “Very deficient” for DPD activity ≤25% wild-type DPYD, “Moderately deficient” for DPD activity within 25–60% that of wild-type, and “Slighly deficient” for DPD activity >60% that of wild-type. Otherwise, variant functionality was considered benign (not statistically significant) or proficient when significantly greater than that of wild-type.–means that the variant was not tested *in vitro*.

MAF means minor allelic frequency, expressed as a percentage.

**Fig 2:**
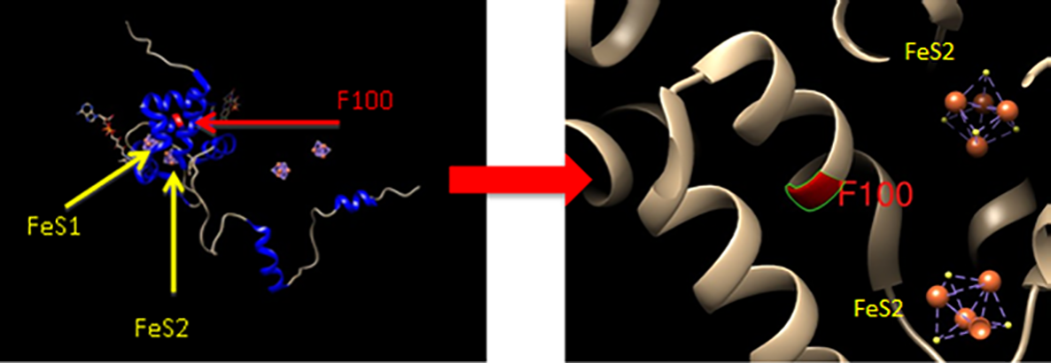
Location of F100L variant within the N-terminal Fe-S cluster containing the alpha helical domain I of the DPD protein. Protein modeling was performed using UCSF Chimera version 1.8. The pig crystal structure (PDB ID 1gTH) was used as a template. The F100L variant could impair enzyme function by disrupting a conserved residue F100 important for electron transfer via the [4Fe-4S] cluster.

* Impact of each *DPYD* variation on phenotype (UH2/U or U) was tested by means of non-parametric Mann-Whitney test for variants present in at least 3 patients (see Statistics section and Fig 2 for details).

** Impact of each *DPYD* variation on digestive/hemato/neurotoxicity (grade 3-4-5 or grade 4–5) was tested by means of Fisher Exact test for variants present in at least 3 patients (see Statistics section).

*** Patients bearing variant allele C29R significantly experienced less toxicity than wt patients (p = 0.041).

^**#**^ Variant linked to haplotype B3 comprising synonymous variant E412E and three intronic variants c.483+18G>A, c.680+139G>A and c.959-51T>C.

ESE means exonic splicing enhancer; ESS means exonic splicing silencer.

nt means not tested due to scarcity of variant carriers (less than 3 patients bearing at least one variant allele).

na means that DPD phenotype was not available (lack of validated UH2/U or U plasma concentration).

NS means not significant (p≥0.05).

Yes means that a significant relationship (p<0.05) was observed (see details in the Results section).

A total of 65 *DPYD* variants were identified: 2 SNPs and one INDEL deviated from the Hardy-Weinberg equilibrium (p<0.07) and 8 SNPs showed poor quality scores or insufficient depth reading. In total, 54 variants (48 SNPs, 6 INDEL) were validated (mean call rate 98.9%). Fifteen variants were located at intron 5 (with one SNP and one insertion at same locus 98185720). Minor allele frequencies (MAF) ranged from 0.2% to 39.7% and were close to those already reported in Exome Variant Server (build Exome Sequencing Project 6500SI-V2-SSA137) or HapMap3 (CEU population) databases. The number of variant alleles per patient varied from 0 (8 patients) to 16 (median 3, mean 4, Q1-Q3: 2–5).

In total, 17 novel variations not identified in the dbSNP 141 database were observed (11 SNPs and the 6 INDELs, with MAF comprised between 0.2% and 2.1%). The six observed INDEL variations were all located in flanking intronic regions (intron 1, 5 and 13). The location of the 48 SNPs was as follows: 3 SNPs in 5’UTR; 19 SNPs in coding regions (4 synonymous including E412E and 15 missenses, including previously reported D949V, V732I, R592W, I560S, I543V, S534N, S492L, M406I, D342G, M166V, T65M, C29R and 3 novel variations A26T, F100L, R696H, each observed in one heterozygous patient); 19 SNPs in flanking intronic regions (including *2A, with no additional splicing variant) and 7 SNPs in 3’UTR. Consensual SNP *2A, D949V and I560S were carried by 7 patients (2.9%, all heterozygous). The location of the 19 observed exonic variants relative to DPD protein structure is shown in **S1 Fig**. Of note, F100L was located within the N-terminal Fe-S cluster containing the alpha helical domain I (**Fig 2**). No large intragenic deletion in the *DPYD* gene was observed by multiplex ligation-dependent probe amplification in the 30 patients with G3-4 toxicity (unshown data).

Analysis of pairwise linkage disequilibrium (LD) between the 52 bi-allelic variants (located at different loci) showed that mainly 2 regions were prone to LD: one restricted to 3’UTR and a second large region ranging from intron 5 till exon 11 (**S2 Fig**). This second region included c.483+18G>A and E412E that composed haplotype B3 (HapB3), along with 5 other linked SNPs (c.483+837A>G, c.483+1342T>A, c.483+1344T>A, M166V, c.1129-15T>C). HapB3 inference was highly likely for the 4 patients bearing E412E variant since they also exhibited the c.483+18G>A variation (all heterozygous).

For the 19 exonic SNPs, results of *in silico* pathogenicity (deficiency) prediction showed 7 variants predicted as pathogenic (including novel variants A26T and F100L), 3 as probably pathogenic (including novel variant R696H), and 9 as benign or probably benign (**Table 3**). **Table 3**also depicts *in vitro* functionality reported in the literature [15,16] for known missense *DPYD* variations. D342G was not tested *in vitro* but codon 342 variation D342N was very deficient *in vitro*. Codon 100 variation F100[FS] was previously associated with loss of enzyme activity *in vitro*. A discrepancy was observed between *in silico* and *in vitro* functionality for variant T65M (proficiency not assessable by means of the UMD-Predictor tool).

### Association between *DPYD* variants and DPD phenotype

**Fig 3:**
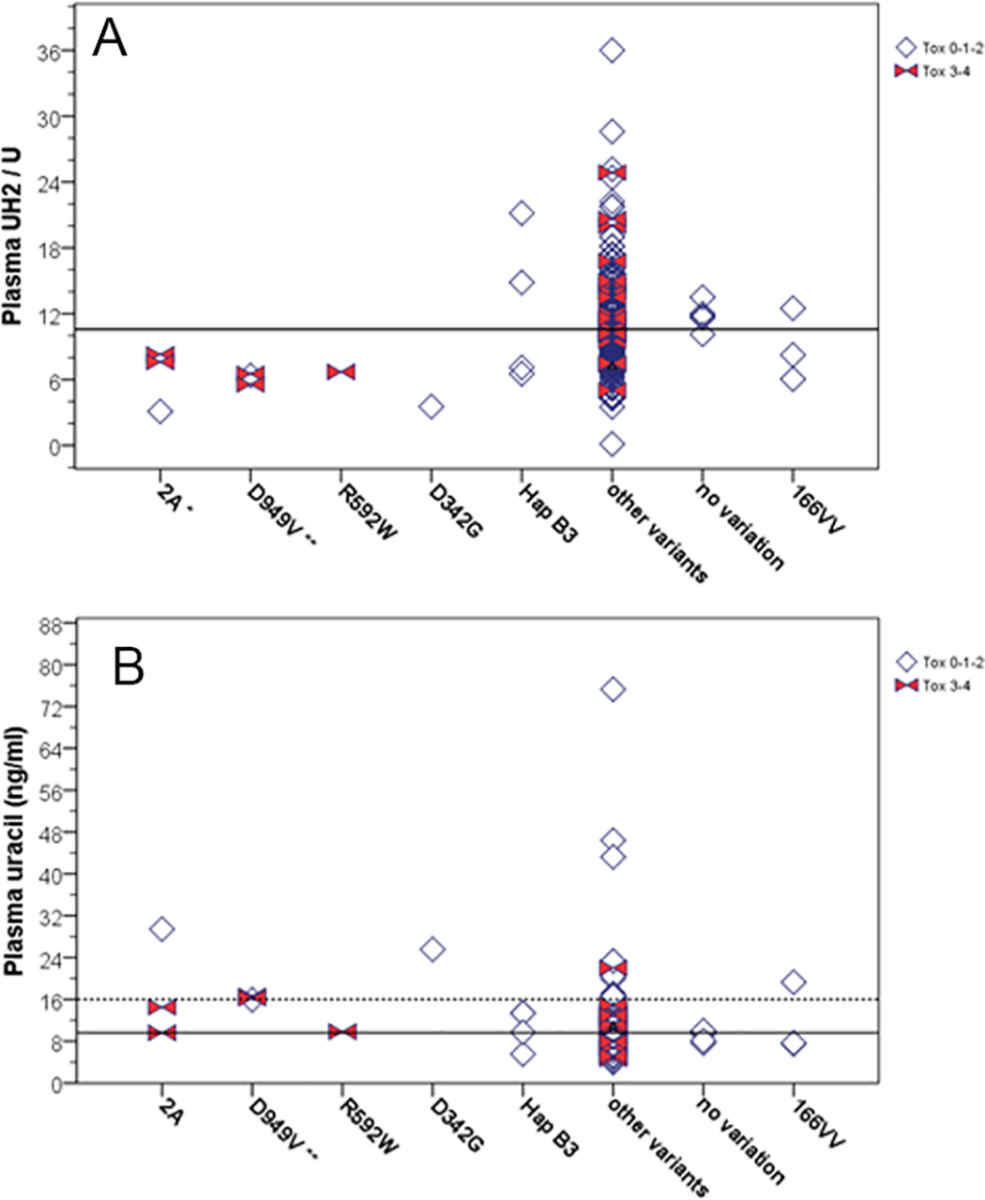
Distribution of pre-treatment plasma UH2/U ratio (A) and Uracil concentrations (B) for the 205 patients with validated phenotypic data, according to *DPYD* variants of interest: variant *2A (3 heterozygous patients), D949V (3 heterozygous patients), R592W (1 heterozygous patient), D342G (1 heterozygous patient), HapB3 (4 heterozygous patients), 166VV (3 homozygous patients) *vs* any other variations (185 patients) *vs* no variation (5 patients). DPD deficiency is reflected by plasma UH2/U decrease or plasma uracil increase. All indicated genotypes were mutually exclusive. Horizontal solid lines indicate median values (10.6 for UH2/U and 9.6 ng/ml for Uracil concentration). Horizontal dotted line on Uracil plot indicates the 91st percentile (16 ng/ml) associated with enhanced grade 3–4 toxicity. Open diamonds indicate patients with toxicity grade 0-1-2 and solid bow ties indicate patients with grade 3–4 toxicity. For variants carried by at least 3 patients, distribution of phenotype was compared between carriers and non-carriers using the non-parametric Mann-Whitney test (* indicates 0.01≤p<0.05 and ** indicates p<0.01).

For the 205 patients with validated phenotypic data, mean pre-treatment plasma UH2/U ratio was 11.1 (median 10.6, Q1-Q3 8.2–12.9, range 0.1–36) and mean plasma U was 10.9 ng/ml (median 9.6, Q1-Q3 7.7–12.2, range 3.9–75.3). The sum of variant alleles did not impact UH2/U or U concentrations. **Fig 3**illustrates the distribution of UH2/U and U plasma concentrations according to *DPYD* variants with very deficient (R592W, *2A, related-D342**N**) or moderately deficient (D949V) enzyme activity *in vitro*, as well as M166V and HapB3. Only variant *2A and D949V were associated with a low UH2/U ratio (p = 0.039 and 0.008, respectively). Only variant D949V was associated with an elevated U concentration (p = 0.005).

### Association between *DPYD* variants and toxicity

Analysis of the impact of each individual *DPYD* variant on toxicity showed that only variant D949V and *2A were significantly associated with an increased risk of developing grade 3–4 toxicity, with similar performance level: 66.7% (2/3) toxicity in wt/var patients *vs* 11.8% (28/239) in wt/wt patients (sensitivity 6.7%, specificity 99.5%, RR 5.69, 95%CI 2.38–13.6, p = 0.041). D949V and *2A variants had no significant impact when focusing on grade 4 toxicity. Of note, the only patient carrying the S492L allele and the patient carrying the F100L allele, both developed grade 4 toxicity (**Table 2**).

Predictive value of *DPYD* variants in combination was next examined (**Table 4**). The presence of one deficient allele among consensual variants *2A, D949V or I560S was significantly associated with grade 3–4 toxicity (sensitivity 16.7%, PPV 71.4%, RR 6.71, 95%CI 3.7–12.2, p<0.001) but was not predictive of grade 4 toxicity. We then considered the 11 *in silico* pathogenic or probably pathogenic variants (*2A, *13, D949V, A26T, T65M, F100L, D342G, G366G, S492L, R592W, R696H). All these variants had very low MAF (<1%) and were only observed in heterozygous state. This set of 11 *in silico* deleterious variants was significantly associated with both grade 3–4 (sensitivity 26.7%, PPV 53.3%, RR 5.48, p<0.001) and grade 4 hemato-digestive-neurotoxicity (sensitivity 60%, PPV 20%, RR 22.6, p = 0.002, **Table 4**). Finally, we considered together variants *2A, D949V and I560S associated with all variants having shown very deficient (<25%) or moderately deficient (25–60%) *in vitro* enzyme activity relative to wild-type (i.e. S492L, R592W, and related-variations D342G and F100L). Taking into account the 7 above-cited *in vitro* deleterious *DPYD* alleles (all mutually exclusive) improved the performance of the genotyping test relative to both the 3 consensual variants and the 11 *in silico* deleterious variants, on both grade 3–4 (sensitivity 26.7%, PPV 72.7%, RR 7.6, p < .001) and grade 4 toxicities (sensitivity 60%, PPV 27.3%, RR 31.4, p = 0.001, **Table 4**).

**Table 4:** Association of variant combinations and/or DPD phenotype with capecitabine-related toxicity (maximum toxicity grade considering hematotoxicity, digestive and neurotoxicity).

Tested biomarkers	Patients at risk	Grade 3–4 toxicity							Grade 4 toxicity						
	/ N total	Sens.	Spe.	PPV	NPV	RR (95%CI)	N event	p ^#^	Sens.	Spe.	PPV	NPV	RR (95%CI)	N event	p ^#^
Three consensual variants (*2A, I560S, D949V)*	2.9% (7/242)	16.7%	99.1%	71.4%	89.4%	6.71 (3.69–12.2)	12.4% (30)	**<0.001**	20%	97.5%	14.3%	98.3%	8.39 (1.07–65.7)	2.1% (5)	0.14
Seven *in vitro* deleterious variants**	4.6% (11/241)	26.7%	98.6%	72.7%	90.4%	7.60 (4.44–13.0)	12.4% (30)	**<0.001**	60%	96.6%	27.3%	99.1%	31.36 (5.8–168.9)	2.1% (5)	**0.001**
Eleven *in silico* deleterious variants***	6.2% (15/241)	26.7%	96.7%	53.3%	90.3%	5.48 (2.95–10.16)	12.4% (30)	**<0.001**	60%	94.9%	20.0%	99.1%	22.6 (4.08–125.1)	2.1% (5)	**0.002**
U >16 ng/ml	9% (18/203) ^##^	12.5%	91.6%	16.7%	88.6%	1.47 (0.48–4.45)	11.8% (24)	0.45	66.7%	92%	11.1%	99.5%	20.56 (1.96–215.8)	1.5% (3)	**0.021**
Combined U>16 ng/ml and/or consensual variants*	10.3% (21/203)	20.8%	91.1%	23.8%	89.6%	2.28 (0.95–5.47)	11.8% (24)	0.082	66.7%	90.5%	9.5%	99.5%	17.33 (1.64–183.1)	1.5% (3)	**0.029**
Combined U>16 ng/ml and/or *in vitro* deleterious variants**	10.9% (22/202)	25.0%	91.0%	27.3%	90.0%	2.73 (1.21–6.14)	11.9% (24)	**0.030**	66.7%	89.9%	9.1%	99.4%	16.36 (1.55–173.2)	1.5% (3)	**0.032**

* The number of patients developing grade 3–4 toxicity among patients carrying *DPYD* variants was 2/3 for variant *2A, 1/1 for variant *13, 2/3 for variant D949V, 1/1 for variant F100L, 0/1 for variant D342G, 1/1 for variant S492L, 1/1 for variant R592W. All these variants were mutually exclusive.

** *In vitro* deleterious variants were *2A, I560S, D949V, F100L, D342G, S492L R592W (See Table 3 for details and literature references).

*** *In silico* deleterious variants were *2A, I560S, D949V, A26T, T65M, F100L, D342G, G366G, S492L, R592W, R696H (see Table 3 for details).

^# ^p value of the Fisher Exact test.

^## ^on this subset of 203 patients, association between either the presence of one variant among the 3 or 7 deleterious *DPYD* variants and grade 3–4 toxicity was confirmed (p = 0.002 and 0.001, respectively) but association with grade 4 toxicity was not.

Sens means sensibility (% of patients positive for the tested biomarker among those experiencing toxicity), Spe means specificity (% of patients negative for the tested biomarker among those without toxicity), PPV means positive predictive value (% of patients experiencing toxicity among those positive for the tested biomarker), NPV means negative predictive value (% of patients without toxicity among those negative for the tested biomarker), RR means relative risk (ratio of the toxicity risk in patients positive for the tested biomarker to that in patients negative for the tested biomarker), NS means not significant.

In patients with validated phenotypic data, distribution of UH2/U was not different according to toxicity. In contrast, uracilemia was higher in patients developing grade 4 toxicity relative to patients who did not (Mann-Whitney test p = 0.016). Patients with uracilemia above 14 ng/ml (i.e. 85^th^ percentile = initial hypothesis) were significantly prone to develop grade 4 toxicity (Fisher Exact test p = 0.047), however significance was not reached when regarding grade 3–4 toxicity. Best uracilemia cutoff was 91^st^ percentile: elevated uracilemia above 16 ng/ml was significantly associated with a RR of 20.6 to develop grade 4 toxicity (sensitivity 66.7%, PPV 11.1%, RR 20.6, p = 0.021) (**Table 4**). As compared with genotyping of either 3 consensual variants, or 7 *in vitro* deleterious, or 11 *in silico* deleterious variants, the combined genotype-phenotype approach (uracil > 16 ng/ml and/or the presence of deleterious *DPYD* allele) did not improve toxicity prediction. For instance, with the best combined approach (7 *in vitro* deleterious variants) sensitivity dropped from 26.7% to 25%, PPV from 72.7% to 27.3%% and RR from 7.6 to 2.7 for grade 3–4 toxicity; for grade 4 toxicity sensitivity increased from 60% to 66.7%, and PPV dropped from 27.3% to 9.1% and RR from 31.4 to 16.4 (**Table 4**).

## Discussion

Capecitabine can induce side-effects that not only impair quality of life and treatment efficacy but may also lead to life-threatening toxicity. Large studies have reported that capecitabine can induce 10% to 25% grade 3–4 digestive toxicity and/or hematotoxicity, 3–10% hand-foot syndrome and 0.2% to 0.6% lethal toxicity [12,22,23,32]. The present observational prospective study conducted on 243 breast cancer patients receiving capecitabine (monotherapy in majority) showed one lethal toxicity (0.4%), 12.4% grade 3–4 hemato/digestive/neurotoxicity and 9.5% grade 3 hand-foot syndrome, in line with literature data. For decades, DPD deficiency has been shown to be the main cause of severe and lethal fluoropyrimidine-related toxicity. Various analytical approaches have been developed for DPD-deficiency screening [33], including direct (PBMC enzyme activity) or indirect (measurement of pyrimidine metabolites) phenotyping, or *DPYD* genotyping. However, a still open question is how to faithfully identify patients at risk of toxicity. Our goal was to analyze exhaustive exome *DPYD* variations and examine their possible relationships with capecitabine-related toxicity and DPD phenotype assessed by pre-treatment plasma U or UH2/U concentrations, considered as a surrogate marker of DPD enzyme activity [29]. To our knowledge, this study is so far the largest prospective one reporting full *DPYD* exome sequencing in patients receiving fluoropyrimidine.

In total, *DPYD* sequencing revealed 54 variants, of which 19 exonic variations including 15 missenses (**Table 3**). *In vitro* functionality has already been reported for 11 presently-observed missense variations, with conflicting results for M166V variant only [15,16]. These two *in vitro* studies reported significantly severe reduced enzyme activity (≤25% relative to wild-type) for variants S492L, I560S, and R592W, moderate reduced activity (25–60% relative to wild-type) for variant D949V, while other variations showed either slight deficiency (>60% relative to wild-type) or no non-functional impact [15,16]. In line with *in vitro* data, variants I560S, R592W and S492L were predicted as pathogenic, and D949V was predicted as probably pathogenic, *in silico*. The presently-observed D342G variation has never been tested *in vitro* but D342N has been associated with very deficient enzyme activity [15]. For novel variant F100L, related in-frame 3-nucleotide insertion F100[FS] (rs72549301) at codon 100 has been associated with dramatic loss of activity *in vitro* [15]. Accordingly, D342G and novel F100L variations were predicted as pathogenic *in silico*. In total, these seven infrequent *DPYD*-deficient alleles (I560S, S492L, R592W, D342G, D949V, F100L and variant *2A) were observed at heterozygous status and were mutually exclusive in the present patient cohort.

Other *DPYD* variants of potential interest are those involved in Haplotype B3 (HapB3), highlighted as a significant predictor of fluoropyrimidine toxicity in a recent meta-analysis [19]. HapB3 comprised variant E412E, c.483+18G>A, and 2 other intronic variants not presently analyzed (c.680+139G>A, c.959-51T>C) [34]. HapB3 is also in tight linkage with the deleterious deep intronic variant c.1129-5923C>G [35]. Four patients carried the E412E allele (heterozygous) and were also the only ones to exhibit c.483+18G>A variation (heterozygous), strongly suggesting that they carried HapB3. As concerns INDELs, the relevant duplication c.168_175dupGAATAATT in exon 3 (*in silico* pathogenic) identified in a DPD-deficient patient with lethal toxicity [31] was not found in the present patient cohort.

In complement to *DPYD* genotyping, we indirectly analyzed DPD phenotype by measuring physiological plasma U and UH2 concentrations, which is a more widely applicable approach than direct measurement of PBMC-DPD activity. Only *2A and D949V were significantly associated with DPD-deficient phenotype (I560S, S492L and F100L not tested due to lack of phenotype data) (**Fig 3, Table 3**). The single patient bearing R592W variant and the one carrying D342G, both expressed low UH2/U ratios. No clear link between phenotype and genotype emerged for HapB3 (4 heterozygous patients), nor for variant M166V (3 homozygous patients) (**Fig 3**). The patient with the most proficient phenotype (uracilemia 3.9 ng/ml) was heterozygous for variant I543V and for 3’UTR variant rs291592. The most deficient patient (uracilemia 75 ng/ml) was heterozygous for variant C29R, two 3’UTR variants (rs56160474, rs41285690) and one 5’UTR variant (rs61787828).

The main objective of this prospective study was to examine relationships between *DPYD* genotype and capecitabine-related toxicity. Consistent with phenotype data, single variant analyses revealed that only *2A and D949V were significantly associated with an increased risk of grade 3–4 digestive, hemato or neuro-toxicities, in line with literature data [22]. The four HapB3 carriers did not present any trend for increased toxicity (no grade 3–4), in line with the results of a recent large prospective study conducted on 1228 5FU-treated patients [36]. The novel *in silico* pathogenic F100L variant was carried by a single patient who developed a grade 4 hematotoxicity associated with grade 3 neurotoxicity (**Table 2**). Interestingly, codon F100 is a very conserved residue located within the N-terminal 4Fe-4S cluster containing the alpha helical domain I of the enzyme (**S1 Fig**). This cluster plays an important role in the electron transfer responsible for the reduction reaction catalyzed by DPD enzyme (**Fig 2**). This clinical observation, along with *in silico* prediction and *in vitro* data reported for related-variant F100 [FS] [15], strongly suggests that infrequent variant F100L may be the causal origin of severe fluoropyrimidine-related toxicity.

Sensitivity of genotyping considering the 3 consensual variants *2A, D949V and I560S together was 16.7% for grade 3–4 toxicity (RR 6.7, p<0.001) and was not significant on grade 4 toxicity (**Table 4**). In order to perform variant combinations as objectively as possible, two combination approaches were tested, one based on *in silico* prediction using the UMD-Predictor system [27], the other relying on existing *in vitro* functional data reporting DPD enzyme activity in transgenically-expressed missense *DPYD* variants [15,16]. The best performance was observed with the combined seven *in vitro* deleterious variants (**Table 4**). As compared to the 3 consensual variants, adding *in vitro* deleterious variants D342G, S492L, R592W and F100L increased the sensitivity to 26.7% for grade 3–4 toxicity, with a similar RR (7.6, p<0.001). Moreover, the combination of the seven *in vitro* deleterious variants was significantly associated with grade 4 toxicity (sensitivity 60%, RR 31.4, p = 0.001) (**Table 4**). Present results show that extended *DPYD* genotyping to known *in vitro* deficient variants clearly improves the performance of consensual *DPYD* genotyping for pre-emptive identification of patients at-risk to develop severe fluoropyrimidine-related toxicities. An alternative strategy, although probably more time-consuming, may be to perform exome *DPYD* sequencing, and consider *in silico* prediction of coding variations. Even though the present study shows this latter approach to be less effective, both strategies deserve to be validated in a future prospective study.

Previous studies have suggested that pre-treatment plasma UH2/U or U is associated with fluoropyrimidine toxicity [30]. A recent large Dutch study [12] confirmed that pre-treatment plasma U is associated with global severe toxicity in patients receiving capecitabine. In the present patient cohort, pretreatment UH2/U ratio and uracil concentrations were not significantly different between patients with or without grade 3–4 capecitabine toxicity (unshown data). However, patients presenting very high plasma U (>16 ng/ml, 91^st^ percentile) were significantly prone to develop grade 4 toxicity relative to patients with U below 16 ng/ml (sensitivity 66.7%, RR 20.6, p = 0.021, **Table 4**). Finally, identifying at-risk patients based on either deficient phenotype (U>16 ng/ml) and/or deficient genotype did not substantially increase sensitivity as compared to genotype alone, while impairing toxicity predictive value and relative risk (**Table 4**). Present results suggest that the best approach for preventing grade 3–4 toxicity would be extended *DPYD* genotype, while prevention of more severe grade 4 toxicity may be based on plasma uracil only. The weak association between indirect DPD phenotyping (uracil, UH2/U) and grade 3–4 capecitabine-related toxicity may reflect the lack of correlation reported between PBMC-DPD enzyme activity and plasma UH2/U ratio, while a significant but weak correlation was observed between plasma U and PBMC activity [16]. This poor correlation between physiological pyrimidine concentrations and DPD enzyme activity may reflect the fact that, under low physiological U concentrations, DPD enzyme is not saturated, suggesting that only a marked DPD deficiency can impact physiological U and UH2 concentrations. Accordingly, literature data show that, in most cases, lethal toxicity is associated with a markedly deficient phenotype based on physiological pyrimidines in plasma [10,12,31], or enzyme activity [5]. In line, the lethal toxicity observed in the present study occurred in a patient presenting elevated U plasma concentration (above the 91^st^ percentile). These observations fully justify the need to implement DPD-deficiency screening based on indirect or direct DPD phenotyping approaches for preventing lethal toxicities. An alternative possible relevant phenotyping approach is to explore uracil metabolism after administration of a loading dose of uracil that results in temporary DPD enzyme saturation [37,38]. Toxicity prevention of capecitabine-based chemotherapies may be improved by additional biomarkers linked to the regulation of DPD expression, such as miR-27a and more specifically MIR27A rs895819 polymorphism [39], or to polymorphisms in other genes related to capecitabine pharmacology, such as *MTHFR*, *CDA*, *TYMS* or *ENOSF1* [40,41,42] that have been shown to be associated with capecitabine toxicity.

In conclusion, DPD-deficiency is recognized to be a leading cause of severe fluoropyrimidine toxicity [6,17] and a recent prospective study has demonstrated that fluoropyrimidine dose-adjustment based on upfront *DPYD* *2A genotyping (dose reduced by >50% in heterozygous carriers) was feasible, cost-effective, and improved safety [43]. Present results extend our understanding of deleterious *DPYD* variants and emphasize their potential impact on the toxicity of capecitabine-based treatments. A next step would be to establish whether upfront fluoropyrimidine dose-adjustment based on an extended DPD-deficiency screening approach could further improve safety without impairing treatment efficacy, while remaining cost-effective.

## Supporting information

S1 Fig Location of exonic *DPYD* variants relative to DPD protein. (TIF)

S2 Fig Pairwise linkage disequilibria (LD) between bi-allelic *DPYD* variants. (TIF)

S1 Table Description of maximal toxicity grade over cycles 1–2 (CTCAE v3 criteria). (DOC)
