# Supplementary figures and images for: New advances in DPYD genotype and risk of severe toxicity under capecitabine
Source: PLoS One. 2017 May 8;12(5):e0175998. doi: 10.1371/journal.pone.0175998 (PMC5421769; doi:10.1371/journal.pone.0175998)

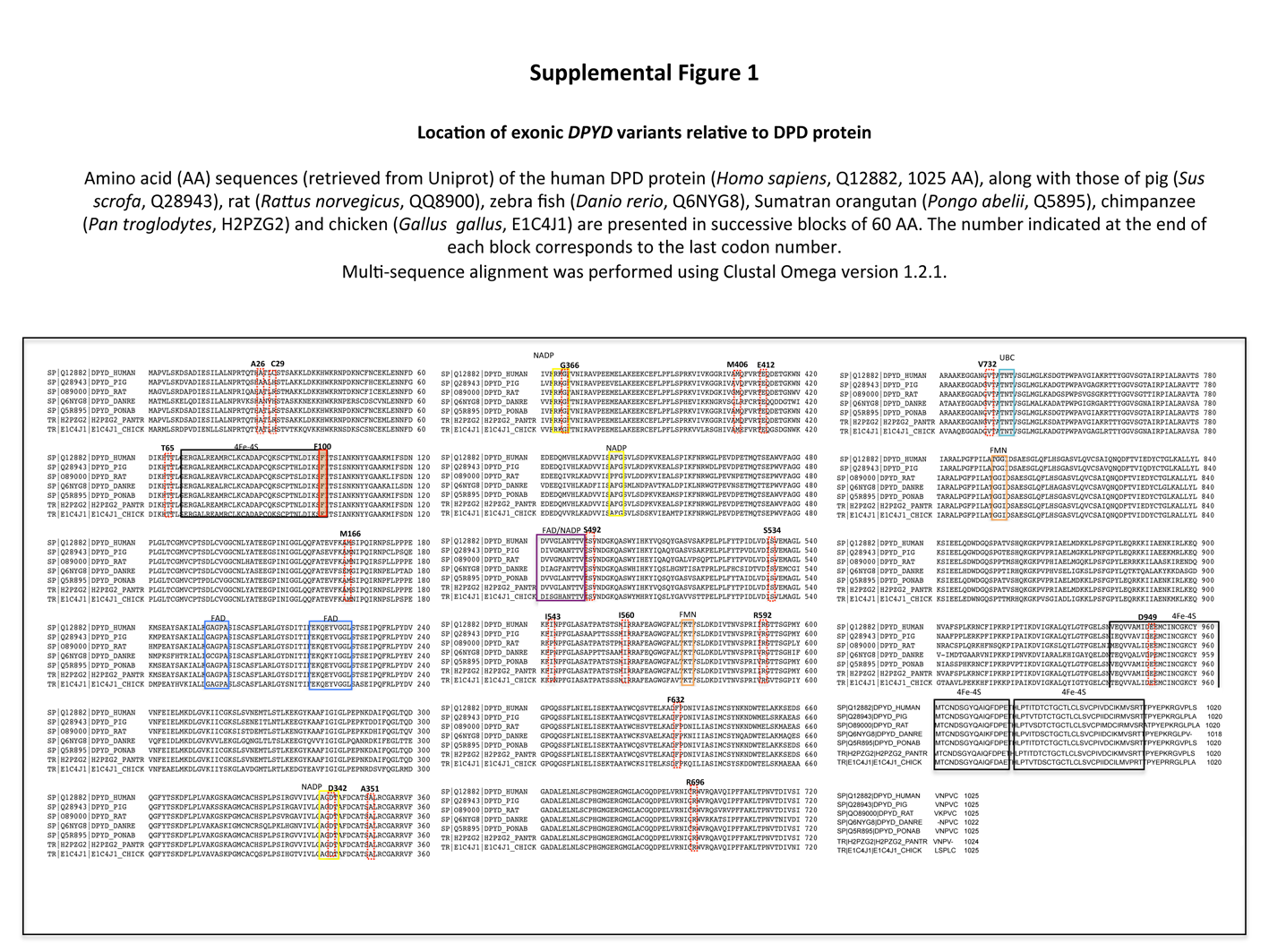

Supplement: S1 Fig — (TIF) [file pone.0175998.s001.tif]

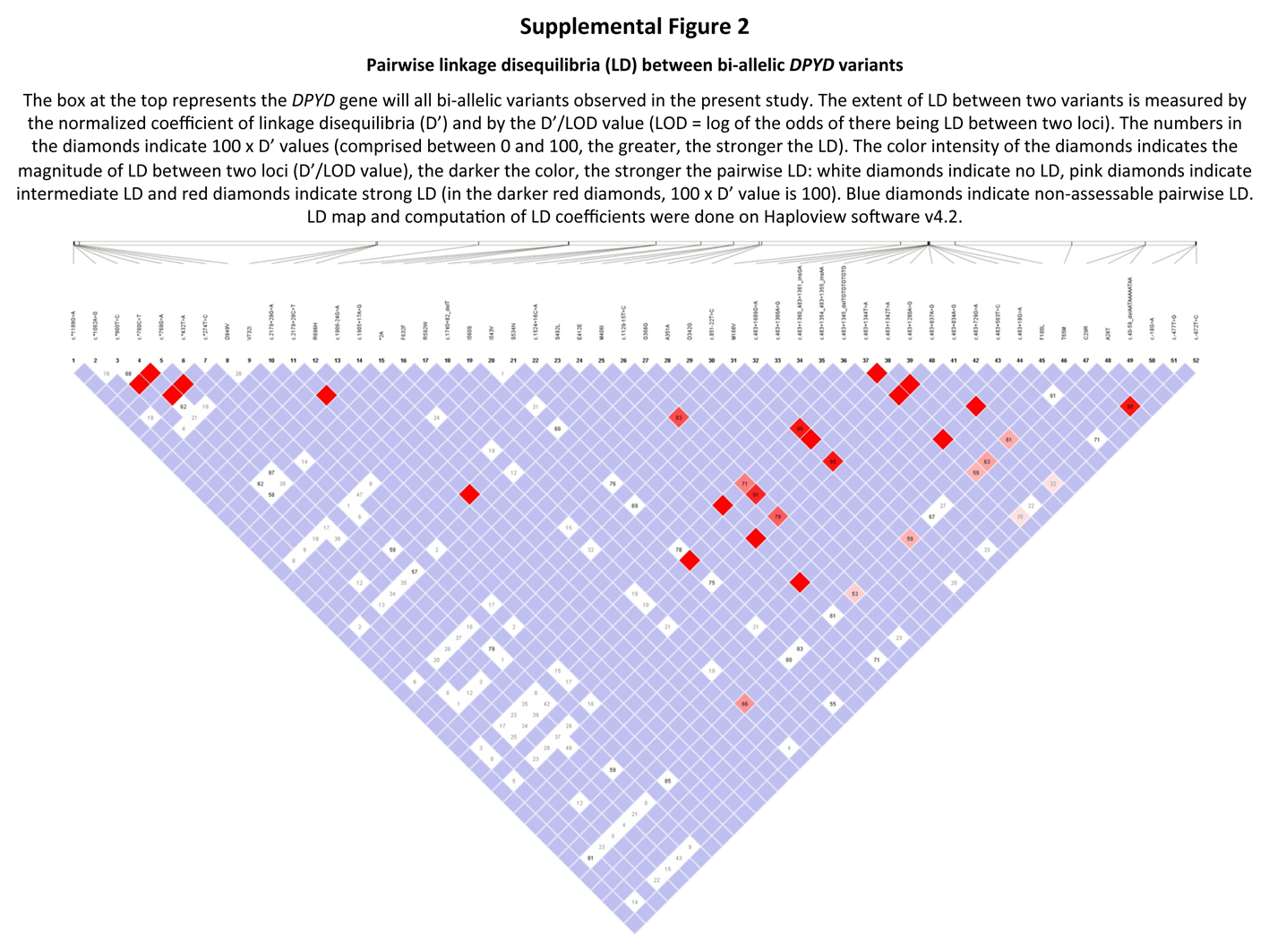

Supplement: S2 Fig — (TIF) [file pone.0175998.s002.tif]
