# Supplementary material for: New advances in DPYD genotype and risk of severe toxicity under capecitabine
Source: PLoS One. 2017 May 8;12(5):e0175998. doi: 10.1371/journal.pone.0175998 (PMC5421769; doi:10.1371/journal.pone.0175998)
Supplement: S1 Table — (DOC) [file pone.0175998.s003.doc]

**Supplemental Table 1**

**Description of maximal toxicity grade over cycles 1-2** (CTCAE v3 criteria)

Number of patients (%)

|  | **Grade 1** | **Grade 2** | **Grade 3** | **Grade 4** |
| --- | --- | --- | --- | --- |
| **Diarrhea**  (N=241)  **Nausea**  (N=240)  **Vomiting**  (N=242)  **Anemia**  (N=239)  **Leucopenia**  (N=238)  **Neutropenia**  (N=239)  **Thrombopenia**  (N=239)  **Neurotoxicity**  (N=242)  **Hand-foot syndrome**  (N=242)  **Cutaneous toxicity**  (N=242)  **Asthenia**  (N=242) | 58  (24.1%)  67  (27.9%)  29  (12.0%)  90  (37.7%)  49  (20.6%)  27  (11.3%)  45  (18.8%)  19  (7.9%)  55  (22.7%)  20  (8.3%)  53  (21.9%) | 13  (5.4%)  19  (7.9%)  6  (2.5%)  15  (6.3%)  12  (5.0%)  19  (7.9%)  2  (0.8%)  5  (2.1%)  40  (16.5%)  12  (5.0%)  35  (14.5%) | 12  (5.0%)  4  (1.7%)  4  (1.7%)  3  (1.3%)  4  (1.7%)  4  (1.7%)  3  (1.3%)  2  (0.8%)  23  (9.5%)  4  (1.7%)  17  (7.0%) | 2  (0.8%)  0  0  1  (0.4%)  1  (0.4%)  1  (0.4%)  3  (1.3%)  0  0  0  0 |
